# Packed red blood cell transfusion associates with acute kidney injury after transcatheter aortic valve replacement

**DOI:** 10.1186/s12871-019-0764-0

**Published:** 2019-06-11

**Authors:** Akeel M. Merchant, Javier A. Neyra, Abu Minhajuddin, Lauren E. Wehrmann, Richard A. Mills, Sarah K. Gualano, Dharam J. Kumbhani, Lynn C. Huffman, Michael E. Jessen, Amanda A. Fox

**Affiliations:** 1 Department of Anesthesiology and Pain Management, University of Texas Southwestern Medical Center, Dallas, TX 75390-8888 USA; 2 Charles and Jane Pak Center for Mineral Metabolism and Clinical Research, University of Texas Southwestern Medical Center, Dallas, TX 75390 USA; 3 Department of Internal Medicine, Division of Nephrology, University of Texas Southwestern Medical Center, Dallas, TX 75390 USA; 4 Department of Internal Medicine, Division of Nephrology, Bone and Mineral Metabolism, University of Kentucky, Lexington, KY 40536 USA; 5 Department of Population and Data Sciences, University of Texas Southwestern Medical Center, Dallas, TX 75390 USA; 6 Department of Internal Medicine, University of Texas Southwestern Medical Center, Dallas, TX 75390 USA; 7 Department of Internal Medicine, Division of Cardiology, University of Texas Southwestern Medical Center, Dallas, TX 75390 USA; 8 Department of Cardiovascular and Thoracic Surgery, University of Texas Southwestern Medical Center, Dallas, TX 75390 USA; 9 McDermott Center for Human Growth and Development, University of Texas Southwestern Medical Center, Dallas, TX 75390 USA

**Keywords:** Blood cell transfusion, Acute kidney injury, Transcatheter aortic valve replacement, Vasoconstrictor agents, Anemia

## Abstract

**Background:**

Acute kidney injury after cardiac surgery significantly associates with morbidity and mortality. Despite not requiring cardiopulmonary bypass, transcatheter aortic valve replacement patients have an incidence of post-procedural acute kidney injury similar to patients who undergo open surgical aortic valve replacement. Packed red blood cell transfusion has been associated with morbidity and mortality after cardiac surgery. We hypothesized that packed red blood cell transfusion independently associates with acute kidney injury after transcatheter aortic valve replacement, after accounting for other risk factors.

**Methods:**

This is a single-center retrospective cohort study of 116 patients undergoing transcatheter aortic valve replacement. Post-transcatheter aortic valve replacement acute kidney injury was defined by Kidney Disease: Improving Global Outcomes serum creatinine-based criteria. Univariate comparisons between patients with and without post-transcatheter aortic valve replacement acute kidney injury were made for clinical characteristics. Multivariable logistic regression was used to assess independent association of packed red blood cell transfusion with post-transcatheter aortic valve replacement acute kidney injury (adjusting for pre-procedural renal function and other important clinical parameters).

**Results:**

Acute kidney injury occurred in 20 (17.2%) subjects. Total number of packed red blood cells transfused independently associated with post-procedure acute kidney injury (OR = 1.67 per unit, 95% CI 1.13–2.47, *P* = 0.01) after adjusting for pre-procedure estimated glomerular filtration rate (OR = 0.97 per ml/min/1.73m^2^, 95% CI 0.94–1.00, *P* = 0.05), nadir hemoglobin (OR = 0.88 per g/dL increase, CI 0.61–1.27, *P* = 0.50), and post-procedure maximum number of concurrent inotropes and vasopressors (OR = 2.09 per inotrope or vasopressor, 95% CI 1.19–3.67, P = 0.01).

**Conclusion:**

Packed red blood cell transfusion, along with post-procedure use of inotropes and vasopressors, independently associate with acute kidney injury after transcatheter aortic valve replacement. Further studies are needed to elucidate the pathobiology underlying these associations.

## Introduction

Aortic stenosis is a common form of degenerative valve disease and its prevalence markedly increases as people age [1, 2]. Many patients with aortic stenosis have comorbidities that place them at higher risk for morbidity and mortality after surgical aortic valve replacement (SAVR). As a consequence, trans-catheter aortic valve replacement (TAVR) has been increasingly utilized as an alternative to SAVR for aortic valve replacement in patients who are intermediate or high risk SAVR candidates. Despite being less invasive than SAVR and not requiring cardiopulmonary bypass (CPB), the risk of TAVR patients developing post-procedure AKI is similar to the risk observed in SAVR patients (~ 10–30%) [3–6]. The occurrence of AKI is associated with increased morbidity and mortality in both SAVR and TAVR patients [3, 7–10]. It is therefore important to identify perioperative risk factors that are potentially modifiable for AKI prevention.

Various clinical risk factors have been associated in observational studies with the development of AKI after TAVR [3, 9–14]. These have included clinical variables such as chronic kidney disease, trans-apical TAVR approach, diabetes, hypertension, peripheral vascular disease, chronic obstructive pulmonary disease, history of myocardial infarction, leukocytosis, bleeding, and blood transfusion [3, 9–14]. Interestingly, a meta-analysis of observational studies reported that contrast media volume is not significantly associated with development of AKI after TAVR [15].

Transfusion of packed red blood cells (pRBCs) during cardiac surgery or TAVR may be avoided depending upon clinical management of factors such as preoperative anemia, perioperative fluid administration, and utilized transfusion thresholds. In cardiac surgical patients, pRBC transfusion has been associated with development of AKI, with perioperative anemia seeming to present an additional additive risk [16–18]. Observational cohort studies examining the association between pRBC transfusion and the development of AKI after TAVR have reported conflicting results [11, 12, 19]. To date, the studies that have assessed the association between pRBC transfusion and AKI after TAVR have not included peri-procedure anemia, fluid balance, intra-procedure hypotension, and intra-procedure and post-procedure inotrope/vasopressor use as potential confounders. Since lower nadir hemoglobin (Hgb), hypotension and need for inotropes or vasopressor drugs may occur in conjunction with bleeding and need for blood transfusion, it is important to assess these factors along with pRBC transfusion in order to identify what risk factors might best be targeted to prevent AKI after TAVR.

Given the potential inter-relations between peri-procedure pRBC transfusion, fluid balance, anemia, hypotension and vasoactive drug administration, this study aimed to assess if pRBC transfusion as well as these other potential risk factors associate with the development of AKI after TAVR. The primary hypothesis of this study is that pRBC transfusion associates with AKI after TAVR even after adjusting for other clinical parameters such as peri-procedure anemia and the use of vasoactive drugs.

## Materials and methods

### Study population

This retrospective single-center cohort study assessed 116 patients who underwent consecutive TAVRs at the University of Texas Southwestern (UTSW) Medical Center from March 20, 2013 to May 11, 2016. The study was approved by the UTSW Institutional Review Board (IRB), and need for patient written informed study consent was waived by the IRB given that this study involved retrospective review of electronic health records (EHRs). Of the 123 patients who underwent TAVR during the study time period, 6 patients were excluded from analysis because of pre-procedure end-stage renal disease, and one patient was excluded from analysis for being an outlier with regards to need for peri-procedure pRBC transfusion (i.e. massive transfusion protocol).

### Data collection

Patient data regarding demographics, preoperative medical history, TAVR device and approach, and intra-procedure and in-hospital post-procedure events were manually extracted from each patient’s electronic health record using a standardized case report form.

### Definitions

The study outcome was the development of AKI within 7 days post-TAVR. Post-TAVR AKI was defined according to Kidney Disease: Improving Global Outcomes (KDIGO) serum creatinine (SCr)-based criteria (i.e. SCr increase by ≥0.3 mg/dl within 48 h after TAVR or an increase in SCr to ≥1.5 times pre-TAVR SCr within the 7 days following TAVR) [20]. Pre-TAVR SCr was defined as the SCr measured before and closest to the time of TAVR procedure. Post-TAVR SCr values were compared with the pre-TAVR SCr for purposes of identifying post-TAVR AKI. Post-TAVR SCr values were evaluated through 7 days after TAVR or until hospital discharge if that occurred earlier than 7 days after TAVR.

Need for pRBC transfusion was assessed during the intra-procedure period and during the first 24 h after TAVR (i.e. peri-procedure pRBC transfusion). Transfusion of other blood products such as fresh frozen plasma, platelets, and cryoprecipitate during TAVR and within the first 24 h post-TAVR were also recorded.

Diabetes was defined as requiring insulin or oral agents. Estimated glomerular filtration rate (eGFR) was defined according to the Modification of Diet in Renal Disease (MDRD) 4 variable equation [21]. Pre-TAVR European System for Cardiac Operative Risk Evaluation (EuroSCORE) II mortality risk was retrospectively calculated by entering clinical data available in the medical record into the calculator found at http://www.euroscore.org/calc.html [22–24]. TAVR procedure duration was defined as minutes between time of vascular access (skin puncture) and the time of post-procedure dressing placement. Type of TAVR device implanted was defined as Generation 1 (Edwards Sapien), Generation 2 (Edwards Sapien XT or Medtronic CoreValve), and Generation 3 (Edwards Sapien S3 or Medtronic CoreValve Evolut).

Pre-procedural anemia was defined by the World Health Organization definition of anemia: hemoglobin (Hgb) < 12 g/dL for women and Hgb < 13 g/dL for men [25]. Nadir Hgb was defined as the lowest Hgb measured as part of routine clinical care during the TAVR procedure or during the first 24 h after TAVR.

Subjects were recorded as being on a preoperative medication if the medication appeared on their preoperative medication list in the electronic medical record. Data regarding timing of last dose before TAVR procedure was not fully available from retrospective review. However, at our institution patients generally do not take angiotensin converting enzyme-inhibitor (ACE-inhibitor) or angiotensin receptor blocker medications during the 24 h before TAVR. Patients generally receive their beta-blocker medication during the 24 h before TAVR.

Maximum number of concurrent inotropes or vasopressor drugs administered was assessed separately for the intra-procedure period and the post-procedure period, as need for transient vasopressor support during the intra-procedure period is not uncommon secondary to vasodilation under anesthesia as well as to facilitate recovery from transiently low cardiac output during valve deployment. However, need for post-TAVR inotrope and vasopressor drugs was prospectively considered by the investigators to represent a persistent need for inotropes and vasopressor drugs that might have greater impact on renal perfusion. The post-TAVR period for which administration of inotrope or vasopressor infusions was assessed included the first 5 postoperative days or until the patient was discharged from the hospital if that was sooner. Inotropes or vasopressor drugs included continuous infusions of any of the following: dobutamine, dopamine, epinephrine, milrinone, norepinephrine, phenylephrine or vasopressin.

Hypotension during TAVR procedure was defined as having at least one intra-procedural episode of ≥5 consecutive minutes of mean arterial blood pressure (MAP) < 60 mmHg. Intraoperative hypotension was assessed for all patients who had continuous blood pressure monitoring via arterial line that was recorded minute to minute in the electronic operating room anesthesia record throughout the TAVR procedure from before induction of anesthesia to the time of patient departure from the operating room at the end of the TAVR procedure. Two patients did not have intraoperative hypotension data available since their charting was done on paper with every 5 min blood pressure noted.

### Statistical analysis

Statistical analyses were performed using SAS (version 9.3; SAS Institute, Cary, NC), and all *P* values were two-tailed with threshold for significance set at *P* < 0.05. Table 1 variables were selected a priori to examine their associations with post-TAVR AKI. Univariate comparisons between patients who did and did not develop AKI were made for clinical and procedural variables using t-tests, Mann-Whitney U tests, Chi-square tests and Fisher’s Exact tests for continuous and categorical data, as appropriate. Multivariate logistic regression was used to assess the association of pRBC transfusion (independent variable) with post-TAVR AKI (dependent variable), with adjustments for pre-procedure estimated glomerular filtration rate (eGFR) as well as those variables in Table 1 with univariate associations of P < 0.05. Number of pRBCs transfused and whether patients were transfused any pRBCs are highly collinear variables, so number of pRBCs transfused was the variable ultimately included in the study’s final multivariable model, since this variable also gives information about transfusion dose.

**Table 1:** Univariate associations between clinical variables and development of acute kidney injury (AKI) after trans-catheter aortic valve replacement (TAVR)

Clinical Variables	No AKI (*n* = 96)	AKI (*n* = 20)	*P* value
Pre-procedure Clinical Characteristics			
Age (years)	81 ± 8	83 ± 6	0.28
Female gender	40 (41.7%)	12 (60.0%)	0.13
Ethnicity			0.76
African American	7 (7.3%)	1 (5.0%)	
White	72 (75.0%)	14 (70.0%)	
Hispanic	11 (11.5%)	4 (20.0%)	
Other	6 (6.2%)	1 (5.0%)	
Diabetes	33 (34.4%)	5 (25.0%)	0.42
BMI ≥ 30 kg/m^2^	25 (26.0%)	2 (10.0%)	0.15
eGFR (ml/min/1.73m^2^)	61 ± 20	52 ± 26	0.10
Serum creatinine (mg/dL; median and IQR)	1.09 (0.89, 1.32)	1.21 (1.00, 1.65)	0.16
Anemia	49 (51.0%)	15 (75%)	0.05
Left ventricular ejection fraction (%) (*n* = 115)	54 ± 12	57 ± 14	0.34
EuroSCORE II (%) (*n* = 115)	6.34 ± 5.67	7.97 ± 6.81	0.26
Pre-procedure Medications			
ACE-inhibitor	40 (41.7%)	8 (40.0%)	0.89
Angiotensin receptor blocker	8 (8.3%)	3 (15.0%)	0.40
Loop diuretic	61 (63.5%)	11 (55.0%)	0.47
Beta blocker	53 (55.2%)	14 (70.0%)	0.22
Statin	74 (77.1%)	12 (60.0%)	0.11
Aspirin	62 (64.6%)	16 (80.0%)	0.18
Procedural Characteristics, Intra- and Post-procedure Events			
TAVR approach			0.61
Transfemoral	73 (76.0%)	15 (75.0%)	
Transapical	13 (13.5%)	4 (20.0%)	
Other	10 (10.5%)	1 (5.0%)	
Contrast volume (mL) (n = 115)	103 ± 48	108 ± 54	0.70
Medtronic device (other device Edwards)	32 (33.3%)	5 (25.0%)	0.47
Generation of device^a^			0.15
1st generation	26 (27.1%)	9 (45.0%)	
2nd generation	44 (45.8%)	9 (45.0%)	
3rd generation	26 (27.1%)	2 (10.0%)	
Rapid pacing	72 (75.0%)	18 (90.0%)	0.14
General endotracheal anesthesia	92 (95.8%)	20 (100.0%)	0.99
Maximum concurrent number of intra-procedure inotropes/vasopressors	1.6 ± 0.8	1.6 ± 0.7	0.79
Occurrence of at least one intra-procedural hypotensive episode; MAP< 60 mmHg for ≥5 mins (n = 114)	39 (41.1)	8 (42.1)	0.93
Total duration of all intra-procedural hypotensive episodes lasting ≥5 mins (mins) (*n* = 114)	8.7 ± 17.6	12.2 ± 20.5	0.45
TAVR procedure duration from initial vascular access (skin puncture) to dressing (mins; median and IQR) (*n* = 111)	115 (97, 144)	133 (105, 180)	0.13
Nadir hemoglobin during procedure and first 24 h post-procedure (g/dL)	9.8 ± 1.7	8.8 ± 1.5	0.02
Total units of pRBC transfused^b^	0.4 ± 0.9	1.7 ± 2.4	0.03
Any pRBC transfused^b^	20 (20.8%)	11 (55.0%)	0.002
Any blood product (pRBC, FFP, platelets, cryoprecipitate) transfused^b^	21 (21.9%)	11 (55.0%)	0.003
Maximum number of concurrent inotropes/vasopressors administered during post-TAVR hospital stay (up to end of post-TAVR day 5)	0.5 ± 0.7	1.1 ± 1.2	0.03

Data are shown as n (%) for categorical variables and mean ± standard deviation for continuous variables unless otherwise noted

^a^ generations of TAVR devices defined as Generation 1 (Edwards Sapien), Generation 2 (Edwards Sapien XT or Medtronic CoreValve), and Generation 3 (Edwards Sapien S3 or Medtronic CoreValve Evolut)

^b^ signifies transfusion during procedure and first 24 h post-TAVR

*AKI* acute kidney injury, *TAVR* trans-catheter aortic valve replacement, *BMI* body mass index, *eGFR* estimated glomerular filtration rate, *ACE* angiotensin converting enzyme, *IQR* interquartile range, *MAP* mean arterial pressure, *pRBC* packed red blood cell, *FFP* fresh frozen plasma

## Results

### Demographic, peri-procedural and clinical characteristics

Table 1 describes clinical and procedural characteristics of the 116 subjects included in the study, with stratification according to whether the patient did or did not develop AKI after TAVR. Twenty subjects (17.2%) developed AKI after TAVR: 19 developed Stage 1 AKI and one developed Stage 2 AKI. No patients developed Stage 3 AKI or required dialysis. Subjects had a mean age of 81 years with a SD of 7.5 years, and 55% of subjects were male. Of the total cohort, 31 subjects (26.7%) were transfused at least 1 unit of pRBCs in the perioperative period. Two patients died within 7 days after TAVR, with one developing AKI prior to death and the other not developing AKI. Post-TAVR ICU stay was significantly longer in the post-TAVR AKI group (median 2, IQR 1, 4 days) versus the group that did not develop post-TAVR AKI (median 2, IQR 1, 2 days) (*P* = 0.02). Post-TAVR hospital stay was also significantly greater in the AKI group (median 5, IQR 3, 12 days) versus the no-AKI group (median 3, IQR 2, 5 days) (*P* = 0.01).

### Univariate associations with development of post-TAVR AKI

Table 1 shows univariate associations between patient demographic, procedural and clinical variables and the development of post-TAVR AKI with data stratified according to whether patients did or did not develop post-TAVR AKI. Patients who developed post-TAVR AKI received significantly more pRBC transfusions than patients who did not (mean units transfused in AKI group 1.7 versus 0.4 units in no AKI group; *P* = 0.03; Table 1). Of the patients who developed post-TAVR AKI, 55% received pRBC transfusion, while only 21% of the patients who did not develop AKI received pRBCs (*P* = 0.002).

**Fig. 1:**
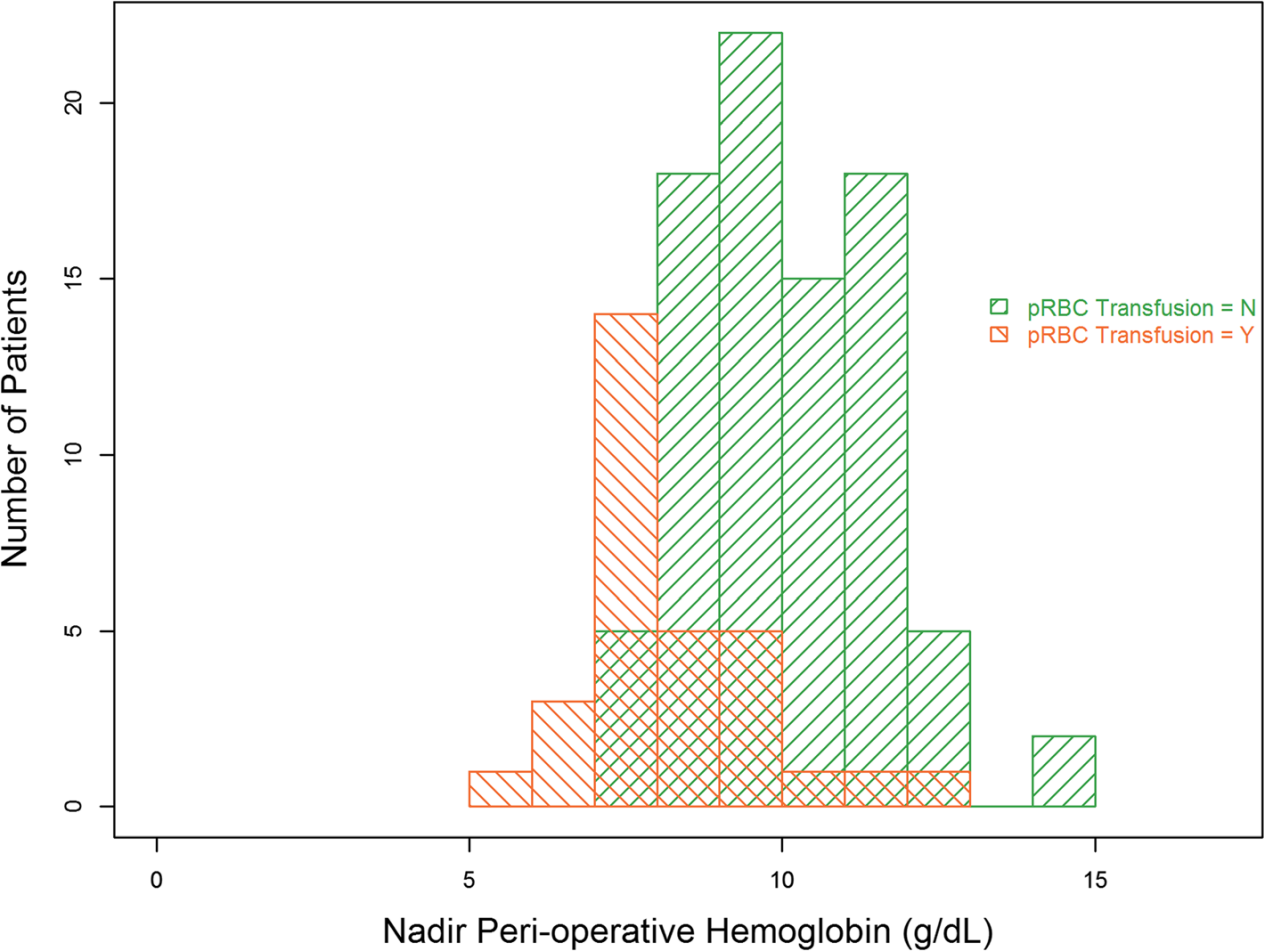
Nadir measured hemoglobin (intra-procedure and first 24 h post-TAVR) and number of patients transfused and not transfused pRBCs at these hemoglobin values. pRBC = packed red blood cells

More patients who developed AKI were anemic prior to TAVR (75% of the AKI group versus 51% of the no AKI group, *P =* 0.05). Furthermore, patients who developed AKI had significantly lower nadir Hgb measurements obtained during the TAVR procedure and the first 24 h following TAVR (mean Hgb 8.8 ± 1.5 g/dL in the AKI group versus 9.8 ± 1.7 g/dL in the no AKI group; *P* = 0.02). Figure 1 shows the patients who did and did not receive pRBC transfusions and the nadir Hgb recorded during TAVR or the 24 h following TAVR for each patient. Since patients may receive pRBCs in the setting of ongoing bleeding and need for volume resuscitation in the operating room, it is possible that some actual nadir Hgb was lower than recorded.

Continuous intra-procedure blood pressure monitoring was done via arterial line. Neither the occurrence of any episode of intraoperative hypotension with MAP < 60 mmHg for ≥5 consecutive minutes, the total number of these intraoperative hypotension episodes, nor the total number of intraoperative minutes included in these hypotensive episodes was significantly associated with development of AKI after TAVR. Having a period of ≥10 consecutive minutes of MAP < 60 mmHg was also not significantly associated with development of post-TAVR AKI. Intra-procedure rapid pacing was utilized in 90% of the patients who developed post-TAVR AKI and in 75% of the patients who did not. This difference was not statistically significant.

Maximum number of concurrent inotropes and vasopressor drugs utilized during the TAVR procedure did not differ significantly between the patients who did and did not develop post-TAVR AKI. However, maximum number of concurrent inotropes and vasopressor drugs utilized during the post-TAVR period (up to hospital discharge or through post-procedure day 5) was significantly greater in the patients who developed post-TAVR AKI than in those who did not develop AKI (mean number of concurrent vasoactive drugs was 1.1 ± 1.2 SD in the AKI group and 0.5 ± 0.7 SD in the no AKI group; *P* = 0.03).

That there is no significant difference in mean contrast volume administered to the patients who did and did not develop AKI (108 mL in the AKI group and 103 mL in the no-AKI group). There was also no significant difference between the AKI and no-AKI groups with regards to percentage of subjects who received greater than or equal to 150 mL of contrast during TAVR.

### Multivariable adjusted associations between pRBC transfusion and development of post-TAVR AKI

**Fig. 2:**
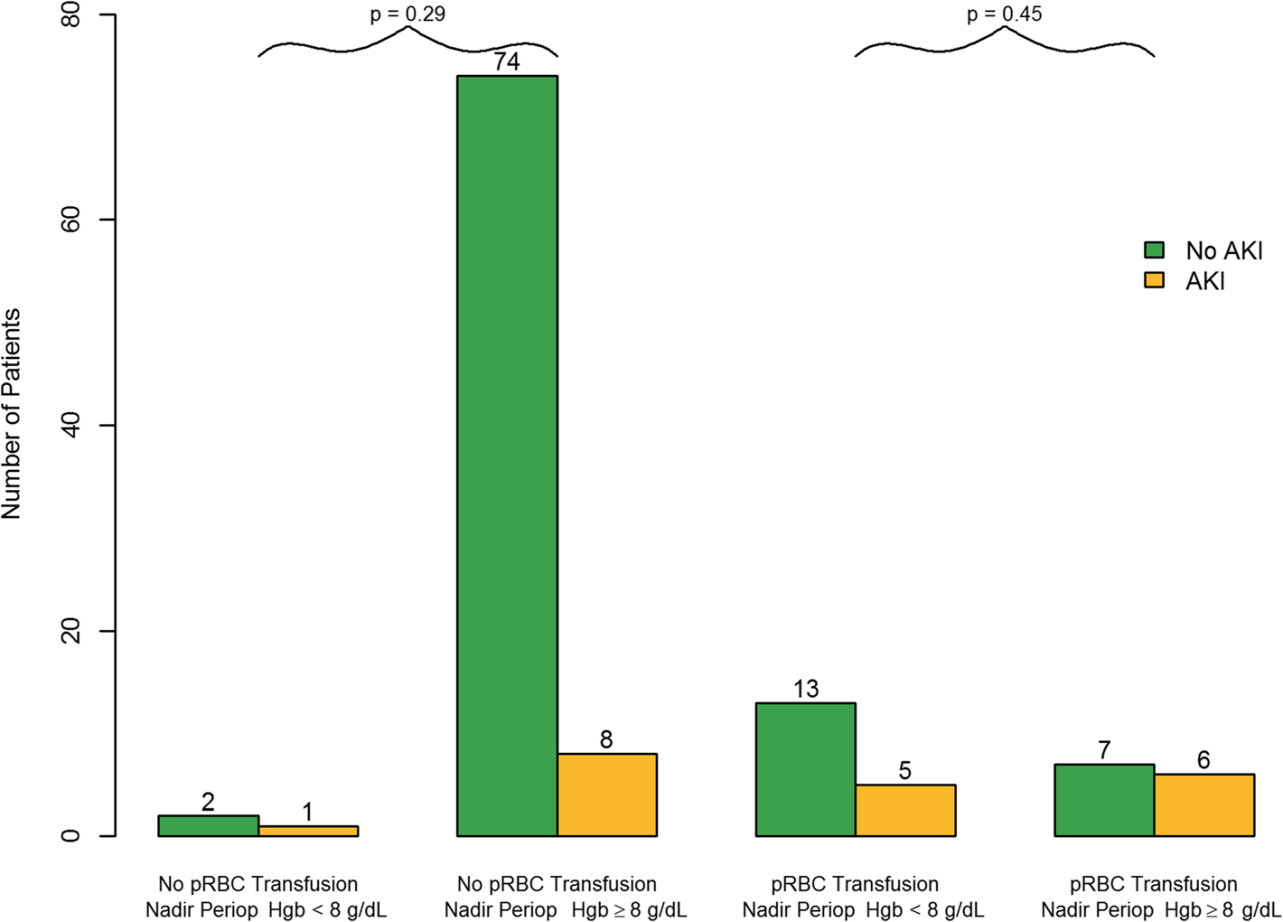
Number of patients with post TAVR acute kidney injury (AKI) stratified by packed red blood cell (pRBC) transfusion and periprocedural anemia (nadir hemoglobin < 8 g/dL versus ≥8 g/dL)

In order to adjust for potential confounders of the association between pRBC transfusion and the development of AKI after TAVR, a multivariate analysis was performed using a logistic regression model with total number of pRBCs transfused, nadir Hgb, pre-procedural estimated glomerular filtration rate (eGFR), and post-TAVR maximum number of inotropes and vasopressors used concurrently (Table 2). Total units of pRBC transfused (OR = 1.67 per unit, 95% CI 1.13–2.47; *P* = 0.01) remained independently associated with post-TAVR AKI after adjustments for these other clinical risk factors. Nadir Hgb was no longer significantly associated with post-TAVR AKI after adjusting for these additional variables. Figure 2 further illustrates the finding that pRBC transfusion rather than peri-procedural nadir Hgb seems to drive the association with post-TAVR AKI; the TAVR study cohort is stratified into 4 groups based on if subjects’ nadir Hgb measured during the TAVR procedure and the 24 h post-TAVR was recorded as < 8 g/dL versus ≥8 g/dL, and then within these nadir Hgb categories patients are stratified according to whether they were or were not transfused pRBCs. AKI development was not significantly different between the patients who were transfused pRBC who had a nadir Hgb of < 8 g/dL and those who were transfused pRBCs and had a nadir Hgb ≥ 8 g/dL (*P* = 0.45). Hgb < 8 g/dL was selected since anesthesiologists typically aim to maintain a Hgb > 7 g/dL and may consider transfusion in the setting of potential ongoing procedural blood loss once Hgb falls below 8 g/dL. Post-TAVR maximum number of concurrently administered inotropes and vasopressors (OR = 2.09 increase for each drug, 95% CI 1.19–3.67; *P* = 0.01) also independently associated with post-TAVR AKI in the multivariable clinical model.

**Table 2:** Multivariable clinical model for predicting development of in-hospital acute kidney injury (AKI) after trans-catheter aortic valve replacement (TAVR)

Clinical Variables	Odds Ratio	95% Confidence Interval		*P* value
Pre-TAVR eGFR (ml/min/1.73m^2^)	0.97	0.94	1.00	0.05
Total units of pRBC transfused during procedure and first 24 h post-TAVR (per 1 unit pRBC)	1.67	1.13	2.47	0.01
Nadir hemoglobin during procedure and first 24 h post-procedure (per 1 g/dL increase)	0.88	0.61	1.27	0.50
Maximum number of concurrent inotropes/vasopressors administered during post-TAVR ICU stay (per each inotrope/vasopressor administered)	2.09	1.19	3.67	0.01

*AKI* acute kidney injury, *TAVR* trans-catheter aortic valve replacement, *eGFR* estimated glomerular filtration rate, *pRBC* packed red blood cell, *ICU* intensive care unit

## Discussion

AKI is known to be associated with increased risk of mortality and renal and non-renal morbidities following TAVR [8, 26]. Despite the less invasive trans-catheter approach versus open SAVR with CPB, 17.2% of the TAVR patients in this study developed post-TAVR AKI. Similar to what has been reported in other TAVR cohorts, most of the AKI observed in this study was Stage 1 AKI [2, 10, 14, 27–29]. However, even Stage 1 AKI is associated with increased mortality in ambulatory [8], non-cardiac surgery [7], cardiac surgery [30] and TAVR patients [27, 28]. Thus, it is important to identify risk factors that can potentially be modified to mitigate development of AKI after TAVR.

In this study we found that pRBCs transfusion during the TAVR procedure or the first 24 h after TAVR significantly associates with the occurrence of post-TAVR AKI. This was true even after adjusting for other clinical parameters including pre-TAVR eGFR, nadir measured peri-procedure Hgb, and post-procedure inotrope and vasopressor use. Just over one-fourth of our TAVR cohort underwent pRBC transfusion. This transfusion rate is similar to or lower than the rates of pRBC transfusion that have been reported in other TAVR cohort studies [10, 14, 29, 31, 32]. Several prior TAVR cohort studies and a recent meta-analysis have also reported significant associations between pRBC transfusion and development of AKI [11, 12, 31, 32]. What differentiates our study from prior studies is that we concurrently assessed the associations between intra-procedure hypotension, peri-procedure anemia, and inotrope/vasopressor usage with occurrence of post-TAVR AKI. These are additional factors that could potentially affect perfusion and oxygen delivery to the kidneys, predisposing to AKI.

There are multiple biologic characteristics of pRBCs and physiologic responses to transfusion that support the concept of pRBC transfusion as a putative cause of AKI. Stored allogenic pRBCs undergo changes in shape and deformability that can decrease oxygen delivery to tissues such as the kidney [33, 34]. Plasma levels of the inflammatory biomarkers bactericidal permeability increasing protein (BPI) and interleukin-6 have also been reported to be significantly elevated in patients who received pRBCs [35]. An in vivo study by Donadee et al identified storage mediated hemolysis causing impaired vascular function due to endothelial dysfunction and vasoconstriction [36]. RBC hemolysis occurs during pRBC storage and following transfusion can lead to increase in plasma free Hgb and iron which cause dysfunction of microcirculation [18].

pRBC transfusion has been associated with postoperative AKI or reduced postoperative eGFR in several observational studies of patients who underwent *cardiac surgery* with CPB [16, 17, 37]. These studies found that preoperative anemia and nadir intraoperative Hgb significantly associate with development of AKI [16, 17, 37]. In our study, nadir Hgb did not remain significantly associated with post-TAVR AKI in the multivariable analysis. Additional larger prospective studies may be warranted to assess for interactive influence of peri-procedural anemia and transfusion in TAVR patients.

To our knowledge no prior study of TAVR patients has assessed the impact of intra-procedure hypotension on development of post-TAVR AKI. One cohort study of 213 TAVR patients did assess the occurrence of any procedural complication that led to severe sustained hypotension and assessed the association between such occurrences and development of AKI. They did not observe an association between such complications and development of post-TAVR AKI, but only 10 patients experienced these complications [32]. We assessed occurrence of MAP < 60 mmHg for greater than or equal to 5 min and also added up the total minutes in all hypotensive episodes that lasted greater than or equal to 5 consecutive minutes. Neither of these intra-procedure hypotension variables significantly associated with AKI in our TAVR cohort. Some have hypothesized that rapid ventricular pacing during TAVR procedure (i.e. period of no blood pressure) may be associated with development of post-TAVR AKI. We did not observe a significant association between rapid pacing and development of post-TAVR AKI, a finding consistent with several other TAVR studies [14, 32].

We assessed hypotension in the intraoperative period but not in the postoperative period, as minute-to-minute post-TAVR blood pressures were not available for our retrospective study cohort. We did find that post-procedure inotrope/vasopressor use was independently associated with development of AKI. Inotrope/vasopressor drug use and its association with AKI has been studied in the cardiac surgical literature with mixed results. A study by Haase et al. did not find an association between vasopressor administration and AKI in patients undergoing on-pump cardiac surgery [37]. A study by Magruder et al. investigating patients who developed AKI following cardiac surgery with CPB showed higher epinephrine dose on ICU arrival and greater total administered dose of epinephrine and norepinephrine in patients who developed postoperative AKI [38]. Porhomayon et al. found a significantly higher incidence of AKI following vasopressin use during coronary artery bypass graft surgery with CPB [39].

It is not uncommon to administer inotropes and vasopressor drugs during the TAVR procedure in order to offset transient effects of anesthesia and/or rapid ventricular pacing. Interestingly, in our study the number of concurrent inotropes and vasopressors administered *during* the TAVR procedure was *not associated* with development of post-TAVR AKI. However, maximum number of required concurrent inotropes or vasopressors administered *after* the TAVR procedure *was an independent predictor* of AKI. This finding warrants further prospective study of the impact of post-TAVR inotropes and vasopressors with a prospectively defined protocol for how inotropes and vasopressors are dosed and when multiple inotropes and vasopressors are administered. Future study also appears warranted to address whether it is post-TAVR hypotension, low cardiac output, or the vasoconstrictive effects of these drugs (or a combination of all of these factors) that predisposes to AKI.

Iodinated contrast media is administered during TAVR procedures and is known to have properties that can cause intense and prolonged vasoconstriction and direct renal tubular damage [40]. However, awareness of the potential toxicity of contrast has led to common practices of pre-procedure hydration, utilization of low osmolar contrast media and minimizing contrast dose. Contrast dose did not associate with AKI in our cohort of TAVR patients. Contrast dose also did not associate with post-TAVR AKI in several other TAVR cohort studies [11, 14, 15, 19, 27–29, 31, 32].

Potential limitations of our study should be considered. It should be recognized that this is a single center observational study, and additional studies at other centers should be performed to validate our study’s findings. The estimated odds ratios for our multivariable model could be biased by the number of AKI events observed in our cohort (*n* = 20), but Vittinghoff and McCulloch (2007) suggest this potential bias is likely minimal [41]. The association between pRBC transfusion and AKI has been observed in some but not all previously reported observational TAVR cohort studies, so our study further corroborates those studies that have reported a significant association between pRBC administration and post-TAVR AKI [12]. However, future studies are needed to work out the pathobiologic mechanism(s) of this observed association. In particular, our findings suggest that anemia may not be a strong modifier of the observed association between pRBC transfusion and post-TAVR AKI. However, our study is an exploratory retrospective assessment, so future larger studies would be useful to validate this observation. Additionally, nadir Hgb was determined from available routine clinical care laboratories. In the setting of bleeding or anticipated bleeding, clinicians might initiate pRBC transfusion before a Hgb level is checked, and, thus, true nadir Hgb might have been missed for some patients. This would possibly underestimate the association between Hgb and AKI. Also, this is a retrospective observational study; therefore a Hgb threshold for initiating pRBCs in clinical practice is not driven by formal study protocol and may have some variability from clinician-to-clinician and patient-to-patient. The significant association between number of inotrope and vasopressor infusions concurrently administered after TAVR and the development of post-procedure AKI is an intriguing finding, but future prospective studies are needed to work out the interactions between patients’ hemodynamic profiles as well as vasoactive drug dosing and the development of AKI in the post-procedure setting. Finally, many of our patients are referred to our medical center for TAVR, but their long-term medical care is continued at outside facilities. Therefore this retrospective study cannot reliably evaluate mid- and long-term post-TAVR outcomes.

## Conclusions

Transfusion of pRBCs but not nadir perioperative Hgb independently associated with post-TAVR AKI. Maximum concurrent number of administered inotropes and vasopressors during the post-TAVR period also independently associated with the development of post-TAVR AKI. Future studies are needed to explore the pathobiological mechanisms underlying these associations as well as to assess the impact of approaches such as pre-procedure anemia optimization and restrictive pRBC transfusion thresholds on the development of post-TAVR AKI.

## Data Availability

The datasets generated and/or analyzed during the current study are not publicly available but are available from the corresponding author on reasonable request and with an approved data use agreement in place between University of Texas Southwestern Medical Center and the requesting researcher’s institution.

## Abbreviations

ACE: Angiotensin converting enzyme
AKI: Acute kidney injury
CI: Confidence interval
CPB: Cardiopulmonary bypass
eGFR: Estimated glomerular filtration rate
Hgb: Hemoglobin
IRB: Institutional review board
KDIGO: Kidney Disease: Improving Global Outcomes
MDRD: Modification of Diet in Renal Disease
OR: Odds ratio
pRBC: Packed red blood cells
SAVR: Surgical aortic valve replacement
SCr: Serum creatinine
TAVR: Transcatheter aortic valve replacement
UTSW: University of Texas Southwestern
